# Bone marrow impairment during early [^177^Lu]PSMA-617 radioligand therapy: Haematotoxicity or tumour progression?

**DOI:** 10.1186/s13550-022-00891-1

**Published:** 2022-04-11

**Authors:** Felix Kind, Kerstin Michalski, Elham Yousefzadeh-Nowshahr, Philipp T. Meyer, Michael Mix, Juri Ruf

**Affiliations:** 1 Department of Nuclear Medicine, Faculty of Medicine, Medical Centre – University of Freiburg, Hugstetter Str. 55, 79106 Freiburg, Germany; 2 German Cancer Consortium (DKTK) Partner Site Freiburg, German Cancer Research Centre (DKFZ), Heidelberg, Germany

**Keywords:** Castration refractory metastatic prostate cancer, PSMA, Radioligand therapy, 177Lu-PSMA-617, Adverse events, Toxicity

## Abstract

**Background:**

The recent phase III VISION-trial confirms the treatment efficacy of radioligand therapy with [^177^Lu]PSMA-617 (PSMA-RLT) in metastatic castration-resistant prostate cancer (mCRPC). In PSMA-RLT, the relatively low absorbed bone marrow dose allows for multiple therapy cycles with relatively low risk of haematological adverse events (hAE). However, as disease progression itself may be a cause of bone marrow impairment, the aim of this study was to assess potential relations between impairment of haematological status and response to PSMA-RLT.

**Methods:**

In this retrospective analysis, haematological parameters (HP) of 64 patients with mCRPC were systematically acquired over two cycles (12–16 weeks) of PSMA-RLT from baseline to restaging. Changes in HP were analysed qualitatively (CTCAE 5.0) and quantitatively. The HP changes from baseline were compared to quantitative and qualitative biochemical and imaging response, using PCWG3 and PROMISE criteria.

**Results:**

All grade 3/4 hAE observed were associated with disseminated or diffuse bone involvement as well as biochemical non-response at restaging. Quantitatively, at baseline, HP inversely correlated with biochemical and volumetric (on PET) tumour burden as well as bone involvement pattern (*p* ≤ 0.043). Among patients with disseminated or diffuse bone involvement, percentage changes in HP (%HP) at restaging inversely correlated with serological and imaging tumour burden (*p* ≤ 0.017). Biochemical non-responders showed a significant decrease in %HP (*p* ≤ 0.001) while HP in biochemical responders remained stable (*p* ≥ 0.079).

**Conclusion:**

During early cycles of PSMA-RLT, qualitative and quantitative bone marrow impairment appears to be closely associated with osseous tumour burden as only patients with advanced bone involvement and non-response to therapy exhibited high-grade haematological adverse events, showing a significant decline of haematological parameters. This implies that in patients with advanced mCRPC, non-response to PSMA-RLT may be a major cause of bone marrow impairment during early treatment cycles.

German Clinical Trial Register DRKS00013665. Registered 28 December 2017, retrospectively registered (www.drks.de/drks_web/navigate.do?navigationId=trial.HTML&TRIAL_ID=DRKS00013665)

## Introduction

Prostate cancer is the second most common cause of all cancer-related deaths for men worldwide [1]. Therapeutic options for advanced metastatic castration-resistant prostate cancer (mCRPC), in particular, are still limited and, thus, survival rates are low [1]. In this setting, radionuclide therapy targeting the prostate-membrane-specific antigen (PSMA), a class II membrane glycoprotein frequently overexpressed by prostate cancer cells [2], is a promising palliative treatment option. [^177^Lu]PSMA-617 radioligand therapy (PSMA-RLT) has shown high treatment efficacy as well as tolerability in both phase II trials [3–5] and the recently terminated phase III trial [6].

Besides the salivary glands and kidneys, bone marrow is known to be a tissue at risk in PSMA-RLT limiting maximal administrable treatment activity and, therefore, potentially efficacy of treatment [7]. Fortunately, PSMA-RLT has shown an absorbed bone marrow dose even lower than [^177^Lu]-DOTATATE [8], with an estimate of 0.012 Gy/GBq [9] well below the estimated tolerance dose of 2 Gy [7], allowing for multiple therapy cycles with relatively low risk of haematotoxicity.

Nevertheless, haematological adverse events (hAE) are witnessed during PSMA-RLT. For example, the VISION and TheraP trials reported severe hAE (grade 3 and 4, as defined by CTCAE 5.0 [10]) in 13% and 8% of patients for anaemia, 8% and 11% of patients for thrombopenia and 13% and 1% of patients for leukopenia, respectively [5, 6].

Just like in peptide receptor radionuclide therapy with Lutetium-177-labelled somatostatin receptor radioligands, bone marrow dose in PSMA-RLT can be considered a result of the dose delivered by marrow-self-absorption and the cross-dose stemming from the remainder of the body- and organ-specific ligand accumulation [7]. Moreover, especially in diffusely metastasised bone tissue an additional crossfire irradiation of haematopoietic bone marrow in the vicinity of osseous metastases has to be assumed as shown by the development of additionally imaging based bone marrow dosimetry protocols [11].

However, aside from this therapy-induced radiation exposure, ongoing displacement of bone (marrow) through metastatic bone involvement [12] in progressive disease has to be considered as another cause of bone marrow impairment and deterioration of haematopoiesis.

This assumption is supported by data on haematological side effects in the control arm of the phase III ALSYMPCA-trial assessing [^223^Ra]Radiumdichloride-treatment of progressive castration-resistant prostate cancer with two or more bone metastases [13]. In said study, when compared to the interventional cohort, a relevant rate of severe anaemia and thrombopenia of 13% (vs. 13%) and 2% (vs. 7%), respectively, was observed in the placebo arm (*n* = 301). Similarly, in mCRPC-patients with diffuse bone marrow involvement, a higher portion of severe hAE during PSMA-RLT seems to be associated with the tumour disease itself [14].

The aim of this retrospective study was, therefore, to systematically determine whether the occurrence of hAE as well as quantitative changes in HP during two cycles of PSMA-RLT is correlated to biochemical and imaging response to therapy.

## Material and methods

### Patient population

Sixty-four patients (median age 74 ± 8.0 years), who had completed at least two full cycles of PSMA-RLT between July 2015 and January 2021, were included in this study. The analysis was approved by the local institutional review board (no.: 251/17). All patients had given written informed consent. Inclusion criterion for retrospective analysis was the availability of complete in-house biochemical and imaging datasets up until restaging after the second cycle. Eligibility criteria used for PSMA-RLT were concordant to recommendations by current guidelines on RLT [15, 16].

### Treatment regime and measurement protocol

PSMA-RLT was administered on a compassionate use base. In accordance with current guidelines [16], either PSMA-617 (*n* = 58) or PSMA-I&T (*n* = 6) was used for radiolabelling with ^177^Lutetium. The radiosynthesis of [^177^Lu]PSMA-617 was done as described previously [17, 18], with slight modifications including the use of n.c.a. [^177^Lu]LuCl3, ammonium acetate buffer and 67 nmol DOTA-PSMA-617. For [^177^Lu]PSMA-I&T, radiosynthesis was further revised with ascorbic acid and ethanol as a scavenger. At least two cycles of PSMA-RLT (at 6 GBq/cycle planned) were applied six to eight weeks apart, followed by biochemical (prostate-specific antigen (PSA)) and PSMA PET/CT-based restaging six to eight weeks later. Depending on response to therapy, PSMA RLT was either continued with two additional cycles, following the same protocol, or discontinued in case of very good response or a clear progression (based on clinical decision).

Haemoglobin concentration (Hb), platelet count (PLT) and white blood cell count (WBC) were monitored from administration of the first cycle (baseline) forward until restaging, weekly (by local oncologist/general practitioner) and centrally on an outpatient base at our hospital every 4 weeks. Serum PSA (Total PSA: Elecsys® total PSA, Roche Diagnostics GmbH, Germany) was ascertained at both baseline and restaging. Tumour volume assessment in all patients was based on whole-body PSMA PET/CT scans acquired before therapy and at restaging. In every individual patient, both PET/CT scans were performed with the same radioligand, either [^68^ Ga]-PSMA‐11 (n = 31) or [^18^F]‐PSMA‐1007 (n = 33). No FDG-based PET/CT scans were conducted. Cumulative bone marrow dose was determined using blood samples and SPECT/CT imaging to quantify cross-irradiation by the remainder of the body (ROB) [19]. Dose caused by bone metastases was not taken into account.

### Changes in haematological parameters and response to therapy

The primary endpoints were frequency of hAE according to the National Cancer Institute Common Toxicity Criteria for Adverse Events (CTCAE, Version 5.0), and quantitative change in Hb, PLT as well as WBC from baseline to restaging. Both were analysed in relation to quantitative and qualitative biochemical (PSA change) and PET-imaging response at restaging after two cycles. This time point was chosen in order to guarantee a homogenous treatment group without, for example, progression-induced therapy dropouts.

Quantitative change in haematological parameters (%HP) at each time point as well as change in PSA (%PSA) and whole-body tumour volume (%TV50) at restaging is expressed as percentage relative to the baseline assessment. Following PCWG3 criteria [20], qualitative change in PSA was categorised as either biochemical response (%PSA ≤ 50%), or non-response (%PSA > 50%). Whole-body tumour volume at baseline and at restaging was determined on PET by semi-automated analysis of PSMA PET/CT acquisitions using percentage thresholding (50% of maximal lesion standardised uptake value) (TV50) as suggested by Seifert et al. [21] using Fiji [22], an ImageJ2-based [23] image processing package, and the Beth Israel plugin for metabolic tumour volume calculation on PET/CT [24]. Metastatic bone involvement on baseline PET/CT was categorised according to PROMISE criteria [25].

### Statistical analysis

SPSS 24 was used for statistical analyses. Data are presented as mean ± standard deviation or range. Univariate and multivariate (Enter method) linear regression was applied to determine possible predictors for haematological parameters at baseline and to correlate change in haematological parameters with biochemical and imaging response, utilising the Akaike Information Criterion for model selection and adjusted *R*^2^ (adj. *R*^2^) for effect size assessment. Difference in haematological parameters over time and between biochemical response groups was assessed using paired or unpaired two-tailed t-test, respectively; Cohen’s d_z_ and Pearson's r were used to estimate effect sizes. Differences in haematological parameters between biochemical response groups at different time points were investigated by one-way ANOVA, utilising ω^2^ for effect size estimation and Tukey post hoc analysis when appropriate.

## Results

At baseline, 35 patients (55%) had previously received at least one line of chemotherapy; 53 (83%), 62 (97%) and 10 (16%) had received radiotherapy, anti-hormonal therapy and [^223^Ra]Radiumdichloride, respectively. On baseline PET/CT, 59 patients (92%) showed bone metastases, with 51 patients (80%) displaying disseminated bone metastases or diffuse marrow involvement (i.e. PROMISE categories miM1b(diss) or miM1b(dmi)). Detailed patient characteristics are given in Table 1. Patients received cumulative mean activity of 11.5 (8.1–14.9) GBq over 2 cycles with a measured mean cumulative bone marrow dose of 0.14 (0.04–0.9) Gy, resulting in an average bone marrow dose of 0.012 ± 0.009 Gy/GBq.

**Table 1:** Patient characteristics at Baseline (*n* = 64)

Characteristic	Data
Age (y)	74 (53–90)
Time since first diagnosis (y)	8.7 (0.7–26.9)
PSA (ng/ml)	324.56 (0.25–3129.00)
Hb (mg/dl)	11.63 (6.10–15.10)
PLT (10^3^/µl)	228.93 (74–422.00)
WBC (10^3^/µl)	5.77 (2.48–10.78)
Bone involvement (*n*)	59 (92)
Uni-/oligometastatic (*n*)	8 (12)
Disseminated-/diffuse (*n*)	51 (80)
TV50 (ml)	107.41 (2.61–877.01)
Previous therapies (*n*)	
Prostatectomy	34 (53)
Anti-hormonal therapy	62 (97)
Chemotherapy	35 (55)
External-beam radiation therapy	53 (83)
[^223^Ra]Radiumdichloride	10 (16)

At restaging, 23 (36%) patients achieved a biochemical response according to PCWG3 criteria, while 41 patients (64%) patients were biochemical non-responders, 56% of which with a biochemical progression (*n* = 23). There were no therapy-related deaths documented in the observation period.

Qualitative data are presented as numbers, followed by percentage in parentheses; continuous data are presented as mean, followed by range in parentheses; bone involvement was categorised according to PROMISE criteria [25]; PSA = prostate-specific antigen, Hb = haemoglobin concentration, PLT = platelet count, WBC; white blood cell count, TV50 = whole-body tumour volume assessed by semi-automated analysis of PSMA PET/CT acquisitions utilising percentage thresholding (50% of maximal lesion SUV).

### Qualitative bone marrow impairment

**Fig. 1:**
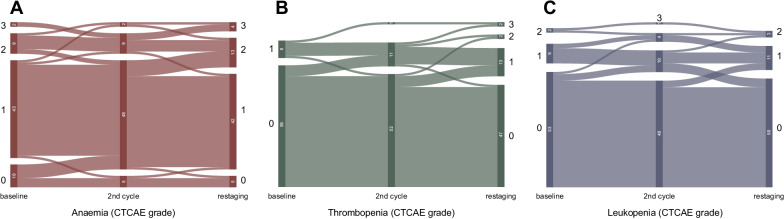
Sankey diagrams for changes in haematological adverse event grades (CTCAE 5.0) among all patients over the observation period (summarised as three time points) for anaemia (**A**), thrombopenia (**B**) and leukopenia (**C**)

Low grade (grade 1 and 2) anaemia was common throughout the observation period, as 52 patients (81%) showed correspondingly lowered Hb already at baseline (Table 2). In addition, lower grade thrombopenia and leukopenia were observed in 9 (14%) and 11 (17%) patients at baseline, respectively. During the observation period, severe (grade 3) anaemia, thrombopenia and leukopenia occurred in 5 (8%), 2 (3%) and 1 (2%) patients, all of whom suffered from disseminated or diffuse bone involvement according to promise criteria and none of whom experienced biochemical response over the course of therapy. Interestingly, the only grade 3 equivalent case of anaemia at baseline was observed in a later biochemical responder who recuperated during the course of therapy. No grade 4 haematotoxicity occurred during the observation period. Changes in hAE grades are further illustrated in Fig. 1. Detailed listings of severe hAE rates are given in Table 3.

**Table 2:** Haematological adverse events during observation period (*n* = 64)

	CTCAE equivalent grading of HP at baseline				CTCAE during 2 cycles of PSMA-RLT			
	Grade 1	Grade 2	Grade 3	Grade 4	Grade 1	Grade 2	Grade 3	Grade 4
Anaemia	43 (67)	9 (14)	2 (3)	0	7 (11)	8 (13)	5 (8)	0
Thrombopenia	8 (13)	1 (2)	0	0	9 (14)	1 (2)	2 (3)	0
Leukopenia	9 (14)	2 (3)	0	0	6 (9)	5 (8)	1 (2)	0

Data are presented as number of patients with percentage of patients in parentheses; CTCAE = Common Toxicity Criteria for Adverse Events version 5.0 [10]

**Table 3:** Rate of severe haematological adverse events during 2 cycles of PSMA-RLT among all patients (n = 64) and different subgroups

Haematological parameter	CTCAE grade 3 during 2 cycles of PSMA-RLT [%] among:			
	All patients (*n* = 64)	a) miM1b (uni/oligo) (*n* = 13)	b) miM1b (diss/dmi) (*n* = 51)	
			biochemical response (*n* = 16)^a^	Biochemical non-response (*n* = 35)^a^
Anaemia	8	0	0	8
Thrombopenia	3	0	0	3
Leukopenia	2	0	0	2

CTCAE = Common Toxicity Criteria for Adverse Events version 5.0 [10], miM1b = bone involvement categorised according to PROMISE criteria [25]

^a^According to Prostate Cancer Working Group criteria v. 3 [20]

### Quantitative bone marrow impairment

Univariate linear regressions were performed in order to identify significant potential predictors for haematological parameters at baseline (Table 4). Ensuing multivariate analysis of these factors revealed that only tumour burden at baseline (PSA_baseline_, TV50_baseline_) as well as the degree of metastatic bone involvement showed an inverse correlation to haematological baseline parameters (see Table 4). There was a significant correlation between biochemical (%PSA) and volumetrical (%TV50) change in tumour burden at restaging (F(1,62) = 5.43, *p* = 0.002, corr. *R*^2^ = 0.29).

**Table 4:** Results of univariate and multivariate linear regressions of various pre-therapeutic variables with haematological parameters at baseline

Linear regression	Variable	Hb_baseline_		PLT_baseline_		WBC_baseline_	
		*p*	adj. *R*^2^	*p*	adj. *R*^2^	*p*	adj. *R*^2^
Univariate	Age	0.371		0.585		0.970	
	PSA	**< 0.001**	0.28	**0.009**	0.11	**0.012**	0.10
	TV50	**< 0.001**	0.32	**0.021**	0.10	**0.037**	0.09
	Bone involvement (PROMISE)	**< 0.001**	0.19	**0.001**	0.17	**0.006**	0.15
	Prostatectomy	0.2		0.132		0.661	
	Anti-hormonal therapy	0.928		0.764		0.850	
	Chemotherapy	0.286		0.376		0.298	
	External-beam radiation therapy	0.58		0.252		0.668	
	[^223^Ra]Radiumdichloride	**0.006**	0.10	**0.014**	0.09	**0.041**	0.08
Multivariate	PSA	**0.004**	0.40	**0.010**	0.23	**0.031**	0.21
	TV50	**0.016**		**0.034**		**0.043**	
	Bone involvement (PROMISE)	**0.029**		**0.002**		**0.011**	
	[^223^Ra]Radiumdichloride	0.173		0.261		0.968	

All variables with statistically significant results (bold) correlate inversely with haematological parameters (Hb, PLT, WBC) at baseline; Hb = haemoglobin concentration, PLT = platelet count, WBC = white blood cell count, PSA = prostate-specific antigen, TV50 = whole-body tumour volume assessed by semi-automated analysis of PSMA PET/CT acquisitions utilising percentage thresholding (50% of maximal lesion SUV)

While HP of 13 patients with no or up to oligometastatic bone involvement (i.e. PROMISE categories miM1b(uni) and miM1b(oligo)) showed no significant decrease in HP (*p* > 0.079), there was a significant mean decrease of Hb (T(50) = 3.59, *p* = 0.001, d_z_ = 0.50), PLT (T(50) = 4.79, *p* < 0.001, d_z_ = 0.67) and WBC (T(50) = 5.05, *p* < 0.001, d_z_ = 0.71) in patients with disseminated or diffuse bone metastases from baseline to restaging.

Among those 51 patients, %PSA correlated inversely with %Hb_restaging_ (F(1,49) = 6.13, *p* = 0.017, adj. *R*^2^ = 0.09), %PLT_restaging_ (F(1,49) = 11.93, *p* = 0.001, adj. *R*^2^ = 0.18) and %WBC_restaging_ (F(1,49) = 7.77, *p* = 0.007, adj. *R*^2^ = 0.10/0.11). Similarly, %TV50 correlated inversely with %Hb_restaging_ (F(1,49) = 6.84, *p* = 0.012, adj. *R*^2^ = 0.11), %PLT_restaging_ (F(1,49) = 7.16, *p* = 0.01, adj. *R*^2^ = 0.11) and %WBC_restaging_ (F(1,49) = 9.63, *p* = 0.003, adj. *R*^2^ = 0.15). The extent of these changes showed neither a significant correlation with cumulative administered activity (*p* ≥ 0.123) nor with cumulative bone marrow dose (*p* ≥ 0.767).

**Fig. 2:**
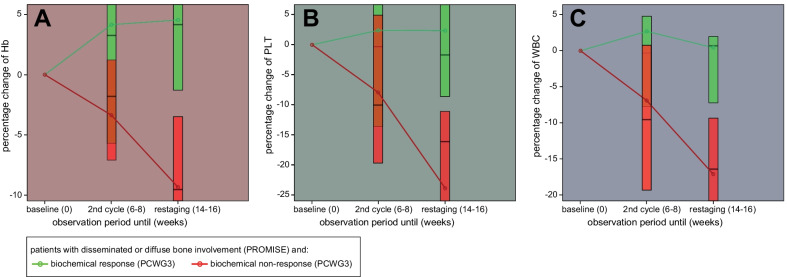
Box plots of percentage changes (in relation to baseline) of haemoglobin (**A**), platelet count (PLT) (**B**) and white blood cell count (WBC) (**C**), at second cycle and at restaging, among patients with disseminated or diffuse metastatic bone involvement (PROMISE) categorised by biochemical response behaviour (PCWG3)

In this subgroup with disseminated and diffuse bone involvement, later biochemical responders and non-responders did not significantly differ in mean Hb (*p* = 0.127), PLT (*p* = 0.081) and WBC (*p* = 0.685) at baseline. However, non-responders showed a progressive decrease of all three parameters over the observation period (*p* ≤ 0.001), while responders remained stable (*p* ≥ 0.458) (Fig. 2). At restaging, the two groups, therefore, significantly differed in all three parameters in favour of the responders. The mean percentage difference was 13.88% [95% CI 5.83–21.92] for %Hb_restaging_ (t(49) = 3.47, *p* = 0.001, *r* = 0.46), 26.37% [95% CI 14.39–38.36] for %PLT_restaging_ (t(49) = 4.42, *p* < 0.001, *d* = 0.55) and 17.61% [95% CI 9.47–25.81] for %WBC_restaging_ (t(49) = 4.34, *p* < 0.001, *d* = 0.55).

Bone marrow dose in responders and non-responders was not significantly different (0.012 Gy/GBq vs 0.011 Gy/GBq, *p* = 0.209), nor did it differ significantly in patients with low and high osseous tumour burden (0.011 Gy/GBq vs 0.012 Gy/GBq, *p* = 0.922). Neither was there a significant correlation between TV50_baseline_ and blood-based bone marrow dose in the first treatment cycle in patients with high osseous tumour burden (*p* = 0.284). Furthermore, the radioligand used for therapy (PSMA-617 or PSMA-I&T) had no significant impact on the changes in haematological parameters at restaging (*p* > 0.458).

## Discussion

In the present study, we assessed the course of haematological parameters (HP) and associated adverse events (hAE) during early PSMA-RLT focusing on their relation to non-responsive disease. This retrospective analysis was done of data derived from compassionate use last-line radiopharmaceutical application [26], thus to a large part including patients with advanced, disseminated or diffuse metastatic bone involvement, a subpopulation to a large part not included in the aforementioned VISION trial [6].

While PSMA-RLT was generally well tolerated in this subpopulation, pathologically lowered HP, corresponding to CTCAE grade one and two, in anaemia especially, were very common among all patients even prior to first application of PSMA-RLT. This emphasises the haematopoietic strain of either preceding therapies and/or the underlying advanced disease itself. Interestingly, all newly occurring severe hAE during PSMA-RLT, while overall small in number, were confined to patients with disseminated or diffuse bone involvement who also displayed a biochemical non-response at restaging (Table 3). The only grade 3 equivalent anaemia associated with a biochemical responder was present at baseline only and improved during therapy with no specific treatment required.

Although CTCAE criteria are very useful for toxicity stratification, it is possible that a small change in HP close to a threshold can result in a shift in category, whereas larger in-category changes will go unnoticed. Therefore, we also analysed the quantitative change of haematological parameters (HP), Hb, PLT and WBC.

At baseline, HP correlated with both biochemical and volumetrically assessed tumour burden on PET as well as bone involvement pattern, while no such correlations were observed for other variables, including previous therapies (Table 4). Furthermore, in line with recent findings reported by Sartor et al. [27], bone-targeting [^223^Ra]Radiumdichloride treatment, was not a significant predicator in multivariate regression analysis for later haematotoxicity, suggesting that the extent of bone metastases is a decisive factor in the haematological behaviour of mCRPC patients. This is also supported by our observation that bone marrow doses were comparable in the subgroups with low osseous tumour burden and those with high osseous tumour burden, irrespective of their treatment response. Neither could a relevant tumour sink effect be observed, as first cycle bone marrow dose and baseline volumetric tumour burden showed no significant correlation.

While HP among patients with either no or few bone metastases remained stable, there was a significant mean decrease of all three HP from baseline to restaging among patients with disseminated or diffuse bone involvement, which inversely correlated with the change in PSA and tumour volume on PSMA PET at restaging, respectively. Further analysis of this subgroup showed that this worsening could mainly be attributed to the biochemical non-responders who showed a significant decrease in all three HP while biochemical responders remained stable (Fig. 2).

Taken together, these observations strongly suggest a close relation between osseous tumour burden prior to, and non-response to PSMA-RLT on the one hand, and qualitative and quantitative decline of haematological parameters on the other hand. These results are comparable to the non-treatment-related high rates of hAE reported in the control arm of the ALSYMPCA-trial [13] and confirm the trend observed by Gafita et al. [14] in their qualitative haematological assessment of patients with diffuse osseous metastases undergoing PSMA-RLT.

Compared to haematotoxicity observed after up to six treatment cycles in the aforementioned phase II and III trials [3–6], our toxicity rates were expectedly lower as we confined our analysis to a shorter observation period of 2 cycles only and cannot be compared on a one-on-one basis.

In this aspect, the low cumulative treatment activities used might in part explain the lack of correlation of changes in HP and bone marrow doses observed, despite the fact that further increasing cumulative treatment activities eventually will induce haematotoxicity as seen in the above-mentioned trials. Therefore, the low likelihood of treatment-related haematotoxicity at this early stage of RLT confirms the assumed causality between haematological status impairment and non-response to PSMA-RLT.

Summarising, in addition to the haematotoxic effect of PSMA-RLT accumulating with an increasing number of cycles, the underlying, non-responsive or even progressive disease itself appears to be an, if not, the important factor for bone marrow impairment in non-responders during early treatment cycles. Potential RLT-induced haematotoxicity must therefore be weighed against definite worsening of haematological status due to disease progression, especially in a last-line setting for patients with advanced metastatic bone involvement, a population largely excluded from aforementioned phase II and III trials.

The present analysis has some limitations. First, it is inherently limited by its retrospective design. Second, in order to assure homogeneity and completeness of data, we confined our analysis of haematological parameters to the first two cycles of therapy until restaging only, thus potentially confining our results to “short-term” toxicity. Nevertheless, we consider this observation period to be sufficient for both the assessment of response, as the largest effect of PSMA-RLT on PSA is expected after the first cycle [4]. Moreover, according to a recent analysis by Gafita et al., the time to platelet nadir for PSMA-RLT can be expected after 3–4 months [14]. Although nadir details on other cell lines after PSMA-RLT are still warranted, data from ^90^Y-radioimmuniotherapy suggest that other blood cell lines seem to have comparable times to nadir [28], which corresponds well with our observation period. Third, imaging response was evaluated using quantitative PET volumetry only according to a recently suggested approach [21], as reliable conventional radiological and PSMA-imaging-based response criteria after RLT in patients with predominantly disseminated bone disease have yet to be defined. Fourth, bone marrow dosimetry was limited to the blood sample measurements and ROB only [19], which tend to underestimate the cross-dose effect derived from bone metastases. On the other hand, as shown by Violet et al. [29], imaging-based bone marrow dosimetry may be limited as well, as in case of diffuse metastases dosages can be overestimated.

## Conclusion

In general, the first two cycles of PSMA-RLT were well tolerated haematologically. Qualitative and quantitative bone marrow impairment appears closely associated with osseous tumour burden as only patients with advanced bone involvement and non-response to therapy exhibited high-grade haematological adverse events as well as a significant mean decline of haematological parameters. This implies that in patients with already advanced mCRPC, non-response to PSMA-RLT may be a major cause of bone marrow impairment during early treatment cycles.

## Data Availability

Raw data were generated at Department of Nuclear Medicine, Medical Centre - University of Freiburg, Germany. Derived data supporting the findings of this study are available from the corresponding author upon reasonable request. The data are not publicly available due to privacy restrictions.

## Abbreviations

CTCAE: Common Toxicity Criteria for Adverse Events
hAE: Haematological adverse events
Hb: Haemoglobin concentration [mg/dl]
HP: Haematological parameters (Hb, PLT, WBC)
%HP: Quantitative change in haematological parameters (Hb, PLT, WBC)
mCRPC: Metastatic castration-resistant prostate cancer
PLT: Platelet count [10^3^/µl]
PSA: Prostate-specific antigen
%PSA: Quantitative change in prostate-specific antigen
PSMA: Prostate-membrane-specific antigen
PSMA-RLT: Radioligand therapy with [^177^Lu]PSMA-617
ROB: Reminder of the body
TV50: Whole-body tumour volume on PET by semi-automated analysis of PSMA PET/CT acquisitions using percentage thresholding
%TV50: Quantitative change in whole-body tumour volume
WBC: White blood cell count [10^3^/µl]
